# Bayesian reasoning with emotional material in patients with schizophrenia

**DOI:** 10.3389/fpsyg.2022.827037

**Published:** 2022-11-03

**Authors:** Verónica Romero-Ferreiro, Rosario Susi, Eva M. Sánchez-Morla, Paloma Marí-Beffa, Pablo Rodríguez-Gómez, Julia Amador, Eva M. Moreno, Carmen Romero, Natalia Martínez-García, Roberto Rodriguez-Jimenez

**Affiliations:** ^1^Department of Psychology, Universidad Europea de Madrid, Madrid, Spain; ^2^Instituto de Investigación Sanitaria Hospital 12 de Octubre (imas12), Madrid, Spain; ^3^CIBERSAM/ISCIII (Biomedical Research Networking Centre for Mental Health/Carlos III Health Institute), Madrid, Spain; ^4^Complutense University of Madrid (UCM), Madrid, Spain; ^5^School of Psychology, University of Wales Bangor, Bangor, United Kingdom; ^6^Facultad de Ciencias de la Salud, Departamento de Psicología, Universidad Rey Juan Carlos, Alcorcón, Madrid, Spain; ^7^Languages and Education Department, Universidad de Nebrija, Madrid, Spain; ^8^CIBERESP/ISCIII (Biomedical Research Networking Centre for Epidemiology and Public Health/Carlos III Health Institute), Madrid, Spain; ^9^Facultad de Ciencias de la Salud, Universidad Francisco de Vitoria, Madrid, Spain

**Keywords:** schizophrenia, emotion, delusions, jumping to conclusions, Bayes theorem, beads task

## Abstract

Delusions are one of the most classical symptoms described in schizophrenia. However, despite delusions are often emotionally charged, they have been investigated using tasks involving non-affective material, such as the Beads task. In this study we compared 30 patients with schizophrenia experiencing delusions with 32 matched controls in their pattern of responses to two versions of the Beads task within a Bayesian framework. The two versions of the Beads task consisted of one emotional and one neutral, both with ratios of beads of 60:40 and 80:20, considered, respectively, as the “difficult” and “easy” variants of the task. Results indicate that patients showed a greater deviation from the normative model, especially in the 60:40 ratio, suggesting that more inaccurate probability estimations are more likely to occur under uncertainty conditions. Additionally, both patients and controls showed a greater deviation in the emotional version of the task, providing evidence of a reasoning bias modulated by the content of the stimuli. Finally, a positive correlation between patients’ deviation and delusional symptomatology was found. Impairments in the 60:40 ratio with emotional content was related to the amount of disruption in life caused by delusions. These results contribute to the understanding of how cognitive mechanisms interact with characteristics of the task (i.e., ambiguity and content) in the context of delusional thinking. These findings might be used to inform improved intervention programs in the domain of inferential reasoning.

## Introduction

Schizophrenia is a chronic and severe disorder manifested through a set of symptoms that can be very disabling, with important impact on personal, familiar and social functioning (Jobe and Harrow, 2005; Tomotake, 2011). Delusions constitute one of the core symptoms of this disorder, but nowadays there are still open questions about its relationship with cognitive processing. Delusions are supposed to rely on a decision-making style called “jumping to conclusions” (JtC), characterized by making firm decisions in the face of limited evidence. It has been found through a study of meta-analysis that this bias is specifically linked to a greater probability of delusion occurrence in psychotic disorders (Dudley et al., 2016). In this sense, there is an ongoing debate about the stability of this bias in reasoning, where delusions might be sustained. Some longitudinal studies suggest that this bias seems to be a stable factor independent of the course of symptoms (Peters and Garety, 2006), while others posit that it can progress, especially during the first years of psychosis (Dudley et al., 2013). A critical feature of delusions is their resistance to be updated in light of conflicting evidence. Hemsley and Garety argued (1986) that delusions precisely occur when a belief is not updated with the new available evidence. A classic task to measure this resistance against updates is the *Beads task* (Phillips and Edwards, 1966). This task allows an evaluation of the process of data gathering in people suffering from delusions (Huq et al., 1988). In the original form of this task, participants are shown 2 jars of beads, one with a higher percentage of beads of one color (e.g., 80% green beads; 20% red beads) and the other with the opposite pattern (80% red beads; 20% green beads). Then, participants watch a sequence of beads apparently being drawn from one of the jars, without specifying from which one. After each draw, participants have to indicate if they are ready to make a decision about which jar the beads came from. This version is known in the literature as Draws to Decision (DtD). Using this version of the task, most of the available literature show that people experiencing delusions (or delusion proneness) usually need fewer beads to make a decision than people without delusions (Ross et al., 2015; McLean et al., 2017). Within this framework, some authors compared performance of participants using different ratios of beads. The logic behind this manipulation is that a ratio of beads of 60:40 is thought to constitute a more difficult-or more cognitively demanding-version of the task, compared to either 85:15 or 80:20 ratios (Ross et al., 2015; Pfuhl, 2017). The 60:40 ratio provides more ambiguous evidence, resulting in low discriminability. Precisely under ambiguous situations the individual’s interpretation plays a fundamental role, compared with scenarios where one option is much more likely than the other (allegedly represented by the 80:20 ratio). For this reason, it is very interesting to study the behavior of patients diagnosed with schizophrenia in both conditions, where the JtC bias may selectively occur under one condition but not the other. If this occurs, it would suggest that this reasoning style is modulated by the prior probabilities of an event. In this sense, patients with delusions usually reach a decision in the presence of less evidence than controls in the 60:40 compared to the 80:20 ratio (Dudley et al., 1997a; Garety et al., 2015). This might be associated with the fact that processing biases become more apparent precisely in ambiguous situations (Combs et al., 2007; Baker et al., 2019). In contrast, other authors have found the opposite pattern: patients requested less information with 85:15 or 80:20 ratios (Dudley et al., 2011; Baker et al., 2019). Some theories point out that patients might improve their performance in presence of more challenging (or more ambiguous) sequences, that will lead them to reduce the JtC bias (Moritz et al., 2007, 2016; Pfuhl and Tjelmeland, 2019). Thus, it is still unclear why conflicting results exist.

In a different version of the task, in which the number of beads drawn from the jar is predefined, participants have to indicate on each trial the probability of the bead being drawn from one jar, in what has been called the Graded Estimates procedure (GE; Huq et al., 1988; Moritz and Woodward, 2004). Using this method, some authors have found that people suffering from delusions exhibited over-confidence in their estimates compared to control subjects (Huq et al., 1988). The GE approach eventually allows to calculate the likelihood of each bead being drawn from a jar using a Bayesian model (Moutoussis et al., 2011). It is thereby possible to study the deviation of participants’ estimates from the exact Bayesian likelihoods (Pfuhl, 2017). Within this framework, Speechley et al. (2010) proposed a version of the Beads task using lakes instead of jars. Patients with delusions were asked to estimate the likelihood that a fish would have been fished from one of the lakes with predominantly black fish (80:20), or another lake with black and white fishes in the same proportion (50:50). Authors found that despite patients selected the lake appropriately; they gave it higher likelihood ratings than controls (Speechley et al., 2010).

Together with data gathering, social information processing is a particular area in which reasoning biases arise in people with delusions (Bentall, 1994; Dudley et al., 1997a). People with delusions preferentially attend to material related to the theme of the delusion (Bentall and Kaney, 1989) which is usually charged with highly affective content (Cannon and Kramer, 2012). Some variations of the Beads task in which positive and negative comments from a simulated survey were presented as beads have been conducted. Results showed that both patients with delusions and healthy controls reduced the amount of information they required to make a decision, compared to their responses in the neutral (original) task (Dudley et al., 1997b). Notwithstanding the importance of the affective content, there have not been many emotionally laden versions of the Beads task. Specifically, the few studies that have considered the emotional content of the stimuli included social material or subjectively meaningful beliefs as beads (see for example Colbert et al., 2010; Lincoln et al., 2011). However, the fact that the emotional material was presented verbally could have made the emotional content less accessible for participants with schizophrenia. Thus, it would be of interest to study data gathering in people with delusions using emotional stimuli that have shown its capacity to highlight possible deficits. In this sense, facial expressions of emotion constitute perhaps the most suitable stimuli to study social information processing (Mandal et al., 1998). Because of that, in the present study we used an emotionally laden version of the beads task including facial expressions of emotions as beads.

Thus, the present study aimed to compare an emotional with a non-emotional version of the Beads task, manipulating the ratio of beads in a within-subjects design. Although few studies have previously included emotional versions of the task, this is, to the best of our knowledge, the first one to include facial expressions of emotion as stimuli. The present study has been conducted using the GE procedure, which enables to calculate the exact deviation of participants from a normative model in order to better understand data gathering processes in schizophrenia. The main goal of this study was to compare performance of patients with schizophrenia suffering from delusions and healthy controls on two versions of the Beads task: the original (neutral) versus one version that included facial expressions of emotions, with ratios of beads of 60:40 and 80:20. Specifically, we intended to study whether responses of patients deviate from the normative model to the same extent in both emotional and neutral tasks. Additionally, we aimed to explore whether deviation scores were related to delusional symptoms.

## Materials and methods

### Participants

Thirty inpatients diagnosed with schizophrenia were included in this study. Patients were recruited from the acute inpatient psychiatric unit at the “University *12 de Octubre”* Hospital, Madrid, Spain. All of them were diagnosed with schizophrenia according to Diagnostic and Statistical Manual of Mental Disorders (DSM)-5 criteria (American Psychiatric Association, 2013) using the Structured Clinical Interview for DSM-5, research version (First et al., 2015), were receiving atypical antipsychotic medication, and were close to be discharged from the acute unit. Clinical status was evaluated using the Spanish version of the Positive and Negative Syndrome Scale (PANSS) (Kay et al., 1987; Peralta and Cuesta, 1994) and the Psychotic Symptom Rating Scales (PSYRATS) (Haddock et al., 1999) (see Table 1 for sociodemographic variables and clinical status). Exclusion criteria for participants included the presence of (a) electroconvulsive therapy in the previous year, (b) neurological disorders or somatic diseases that could interfere with performance on the tasks, (c) active substance dependence (excluding caffeine and nicotine), (d) intellectual disability (IQ below 70), (e) autism spectrum disorder, and (f) inability or unwillingness to complete the psychometric measures at baseline. On the other hand, 32 age and gender-matched healthy controls were recruited from the same geographic and socio-cultural area. None of them had personal or first-degree family history of psychotic disorders. The study protocol was approved by the Ethics Committee of the Hospital (18/333) and written informed consent was obtained from all participants prior to their inclusion in the study.

**Table 1:** Sample demographics and clinical status of patients.

	Patients (*n* = 30)	Controls (*n* = 32)	
	Mean (SD)	Mean (SD)	Statistic (*p*)
Age (years)	37.0 (14.2)	32.3 (10.9)	*t* = −1.45 (*p* = 0.15)
Gender (% male)	46.7%	43.8%	*χ^2^* = 0.053 (*p* = 0.82)
Duration of illness (years)	10.7 (11.8)	--------	--------
PANSS-Positive	19.3 (4.0)	--------	--------
PANSS-Negative	16.7 (5.8)	--------	--------
PANSS-General Psychopathology	36.0 (4.8)	--------	--------
PSYRATS-Hallucinations	15.5 (11.1)	--------	--------
PSYRATS-Delusions	14.7 (3.0)	--------	--------
CPZ^*^	471.6 (271.7)	--------	--------

* Chlorpromazine equivalent dose (mg/day).

### Materials

Scenarios were created using Adobe® Photoshop CC. The facial stimuli included in the emotional version of the task consisted of 32 pictures corresponding to 8 models (4 female, 4 male) taken from the NimStim stimulus set (Tottenham et al., 2009). Two pictures from each model showing happy and angry expressions were selected. Pictures were converted to grey scale, equated in luminance, and cropped to conceal most of the hair to remove distracting, noisy aspects that are not informative of emotional expression (Calvo and Lundqvist, 2008).

### Procedure

**Figure 1:**
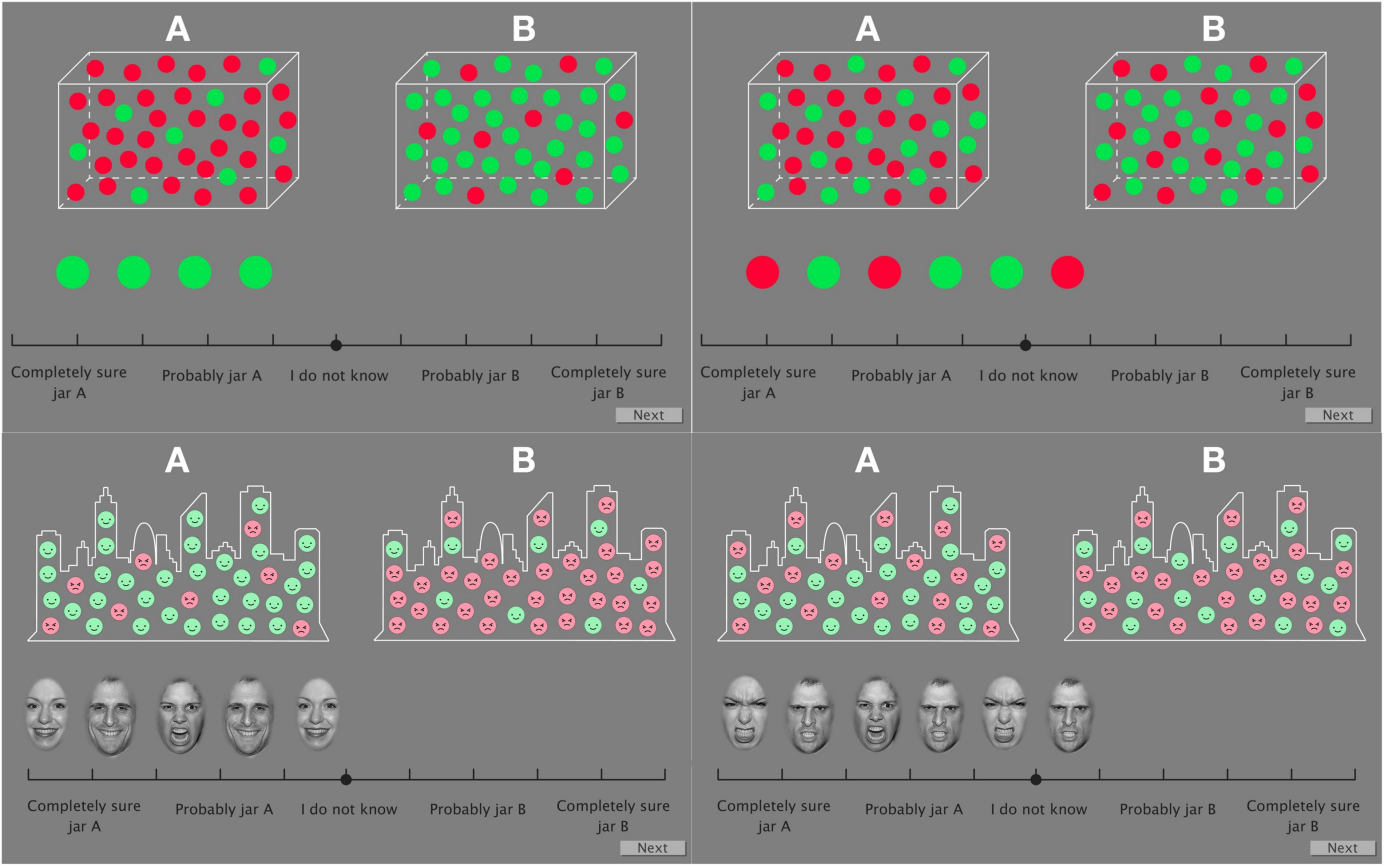
An example of four different trials of the Beads task with neutral (top row) and emotional (lower row) content. Left screenshots show trials corresponding to the 80:20 jar ratio. Specifically, in the top left example, Series 5 (0Red:100Green) is represented; in the bottom left example Series 6 (80Happy:20Angry) is presented. Right pictures show trials of the 60:40 jar ratio. Specifically, top right picture presents Series 1 for beads (60Red:40Green) and Series 5 (0Happy:100Angry) for faces on bottom right side. Images used with permission from Tottenham et al.(2009).

The present study comprised two versions of the Beads task. In the first one, similar to the original task, participants were shown two jars with green and red beads. The first five series were showed in presence of an 80:20 ratio of beads. Each series was composed by 10 beads apparently drawn from one jar. Participants were instructed to rate the likelihood that each bead was being drawn from each jar by clicking with the mouse in a slide bar ranging from “completely sure jar A” to “completely sure jar B.” As a way of controlling the need for memory involvement which has been proven to be impaired in schizophrenia (Lee and Park, 2005), the previous beads were kept visible as other authors have done (Menon et al., 2006). After these, another five series of 10 beads with jars containing a 60:40 ratio started. The second task had the same structure but with emotional content. The scenario was presented as two villages in which “mostly happy people” and “mostly angry people” lived in. In this case, faces of citizens instead of beads were presented. An example of four different trials can be seen in Figure 1. To make both tasks comparable, the series of emotional content were identical to those of the neutral version: every time a red bead appeared on the neutral version, a happy face was presented in the emotional version, and the same for green beads and angry faces.

For each experimental condition (beads/faces in either 80:20 or 60:40 ratios) five series were presented: one following the ratio of jar A, one following the ratio of jar B, two series entirely composed of each color or emotion (100:0 or 0:100) and a series with a 50:50 ratio. Therefore, there were two types of ratios manipulated in this experiment: (1) the Jar ratio of beads belonging to each color (80:20 and 60:40) and (2) the ratio of beads presented into each series (0:100, 80:20, 60:40, 50:50). The 100:0 series is a novelty with regard to previous studies. It was included because it would represent the most conclusive level of evidence in favor of one of the jars and participants’ responses in this series may provide some interesting information. Supplementary Table 1 presents detailed composition of the series.

### Statistical analysis

Data was analyzed using R Studio Version 1.2.1335 (R Core Team, 2013). The coordinate of the slide bar corresponding to each participants’ response, which was the likelihood rating, was converted to a value between 0 and 10 (*Y*). By using this score the “Deviation from the normative model” was calculated. First, the exact probabilities following the Bayes theorem referring to jar B were calculated. The difference between the responses of participants and the normative model constituted the deviation score. Appendix A (Supplementary material) provides details of all the calculations.

This study has a repeated measures design with a between-subjects factor: Group (patients and controls). The within-subjects factors included were Series (with seven levels as shown in Supplementary Table 1), Content (neutral or emotional) and the position of the bead or face. The latter was considered as a numeric variable with values ranging between 1 and 10. Data for each ratio of beads were analyzed separately. All first-and second-order interactions involving the Group factor were studied. An analysis of generalized estimation equations (Liang and Zeger, 1986) was used. The estimation-equation approach of population average models has been postulated as a closer approximation to reality compared to the mixed-models or the basic regression approaches when the independence assumptions are violated (Hubbard et al., 2010). Specifically, the *geeglm* function from the geepack package was used (Halekoh et al., 2006). Model fitting procedures are illustrated in Appendix B (Supplementary material).

Finally, Pearson’s correlations were conducted between clinical measurements of delusions (PANSS-P1 item (delusions) and PSYRATS-D items) and (1) global averages of the 60:40 and 80:20 conditions (all series) separated by content (neutral vs. emotional), (2) scores of the last bead/face of each series (as it is the trial with more available information), also separated by content and (3) averages of the 10 beads/faces of each series, also separated by content.

## Results

As can be seen in Table 1, prior to the analysis, patients and controls were compared in sociodemographic variables (age and sex) using Student’s *t*-test and chi-squared test, respectively. No significant differences between the proportions of men and women (*χ*^2^ = 0.053, *p* = 0.82), nor in the mean age (*t* = −1.45, *p* = 0.15) between patients and controls were found.

### Difference from the “ideal observer”

In the 60:40 ratio, only the factor Group was significant (*χ*^2^ = 13.9, *p* < 0.001). Overall, patient’s deviation from the normative model was 0.93 points greater than controls’, while holding other variables in the model constant.

Regarding the 80:20 ratio, a main effect of Group (*χ*^2^ = 12.3, *p* < 0.001), Series (*χ*^2^ = 123.0, *p* < 0.001), and Content (*χ*^2^ = 13.9, *p* < 0.001) was found. The deviation from the normative model of patients was on average 0.7 points greater than the deviation of controls when the remaining variables of the model are kept constant. The second-order interaction Group*Series*Content was also significant (*χ*^2^ = 16.2, *p* = 0.002). A direct comparison between patients and controls showed that there were differences in all series except for Series 7 (20:80 ratio) when the content was neutral (See Table 2). However, when the content was emotional, differences between groups were restricted to Series 5 (0:100 ratio; *p* = 0.007), Series 6 (80:20 ratio; *p* = 0.04), and 7 (20:80 ratio; *p* < 0.001). In all cases, responses from patients deviated from the normative model to a greater extent than responses from controls.

**Table 2:** Least-squares means and standard errors for the difference from the “ideal observer.”

	Series	Ratio of 80:20			
		Controls	Patients	*p* value	95%CI
Neutral	6	2.0 (0.2)	3.1 (0.4)	**0.009**	[−1.8; −0.3]
	2	1.1 (0.2)	1.8 (0.3)	**0.03**	[−1.4; −0.1]
	3	1.8 (0.1)	2.6 (0.3)	**0.005**	[−1.5; −0.3]
	7	2.2 (0.1)	2.7 (0.2)	0.08	[−1.0; 0.1]
	5	1.5 (0.2)	2.6 (0.3)	**0.001**	[−1.8; −0.4]
Emotional	6	2.0 (0.3)	2.8 (0.3)	**0.04**	[−1.5; −0.1]
	2	1.1 (0.2)	1.5 (0.4)	0.34	[−1.3; 0.4]
	3	4.2 (0.2)	4.1 (0.2)	0.70	[−0.4; 0.7]
	7	1.8 (0.2)	3.0 (0.3)	**<0.001**	[−2.0; −0.5]
	5	1.4 (0.1)	2.2 (0.3)	**0.007**	[−1.4; −0.2]

Significant values (*p* < .05) are highlighted in bold.

Regarding the correlation between patients’ deviation from the normative model and delusional symptomatology, we found a statistically significant correlation between PANSS-P1 and the average of series 1 and 4 (60:40 ratio) in the neutral condition (*r* = 0.40, *p* = 0.03) and the average of series 6 and 7 (20:80 ratio) also in the neutral condition (*r* = 0.46, *p* = 0.01). Furthermore, the PSYRATS-D6 item (disruption to life) significantly correlated with the average of series 1 and 4 (60:40 ratio) in the emotional condition (*r* = 0.39, *p* = 0.04).

## Discussion

The main goal of this work was to study impairments in cognitive processes such as statistical inferential reasoning in patients diagnosed with schizophrenia experiencing delusional ideation. Specifically, we aimed to study the deviation of participants’ responses from the ideal observer model under two types of bead ratio combined with two types of task content. In essence, we found widespread differences between patients and controls in the 60:40 ratio condition, while in the 80:20 ratio condition they are restricted to specific series. Moreover, in the 80:20 ratio, there were significant differences between patients and controls in more neutral series than in the emotional condition.

The first result for the purpose of this study was that patients with schizophrenia were likely to deviate from the exact probabilities to a larger extent than controls, and such deviation increased when the composition of jars had a 60:40 ratio (compared to 80:20 ratio). This result suggest a data-gathering bias that complements what other authors have found using the DtD approach, in which people suffering from delusions request less information to make a decision (Dudley et al., 1997a; Garety et al., 2015). As it was hypothesized, the decision-making style of patients with delusional ideation has become evident under the 60:40 ratio. In this sense, it has been argued that many delusions tend to arise when the perceptual system is dealing with different forms of uncertainty (Hohwy, 2013). This result has striking clinical implications, as it demonstrates that the more ambiguity, the more inaccurate are probability estimations in patients with schizophrenia.

The comparison between patients and controls across series and content in the 80:20 ratio, showed that there were differences in all series except for Series 7 when the content was neutral and Series 5, 6 and 7 when the content was emotional. This could be interpreted as a restriction in the series in which patients and controls differ, as opposed to the overall differences found when the ratio of beads was 60:40. It should be noted that there were more neutral than emotional series in which differences were statistically significant. This is probably because controls increased their distance to the normative model in some emotional series, thus, reducing differences with patients’ responses. This effect might be explained in terms of cognitive demands. Performing a probabilistic reasoning task while processing emotional material involves combining two very demanding types of tasks. Facial stimuli has proven to be a type of “privileged” information to which our processing system quickly allocates cognitive resources (Vuilleumier, 2000). There is neural evidence that emotional information has a detractor effect on cognitive processing of other types of simultaneous material (Dolcos and Mccarthy, 2006). Then, the deviation of healthy controls in some series of emotional content might be explained by the allocation of cognitive resources in decoding emotional features at the expense of probability calculations. There are, however, significant differences in some emotional series, in which patients with schizophrenia show a greater deviation than control subjects from the normative model. In previous studies, a jumping to conclusions bias in the case of patients diagnosed with schizophrenia was found to be greater with emotionally salient material (Young and Bentall, 1997; Dudley et al., 1997b). This effect has also been found even in delusion-prone otherwise healthy individuals (Warman and Martin, 2006; Van Der Leer et al., 2015). It is worth noting that the study by Dudley et al. (1997b) used self-referential material and the study by Young and Bentall (1997) used personality traits as emotional material. Given that verbal descriptions were used, their results are not entirely comparable to ours. Our results, contribute to put in a wider perspective previous published research. Thus, responding to the objectives of the study, we have not found patients to show remarkably higher deviations in the emotional condition. In fact, it appears that in the emotional condition differences between patients and controls decrease, probably because estimations made by both groups move away from the normative model in a similar way.

Finally, the amount of deviation from the normative model in the neutral (original) version of the task (in both 60:40 and 80:20 ratios) positively correlated with severity of delusions as measured with PANSS-P1. More interestingly, the amount of deviation in the 60:40 series with emotional content significantly correlated with disruption to life as measured with PSYRATS-D6. Similarly, some previous authors have found correlations between JtC bias and delusions in schizophrenia (Moritz and Woodward, 2005) and even with delusional proneness in control samples (Van Der Leer et al., 2015). Moreover, other researchers have reported a correlation between severity of psychotic symptoms and JtC specifically in the more demanding version of the task (i.e., 60:40 ratio; Krężołek et al., 2019). However, we found an extremely interesting result, as it appears that patients’ impairments on the task in its original form relates to the presence of delusions, but when the emotional content comes into play in the ambiguous condition, impairments are related to the impact of delusions on their lives. This result opens a new approach to the study of JtC with emotional content-specifically with emotional faces-. Impairments shown by people diagnosed with schizophrenia in this task could be an indicator of not the quantity of delusions, but the way they interfere with their daily lives.

Finally, it should also be stressed that both patients and controls deviated to some extent from the normative model across all conditions. In this sense, it has been argued that prior probabilities are sometimes neglected even in normal reasoning (Tversky and Kahneman, 1974), indicating that human reasoning and, in general decision-making processes are not only based on estimation of probabilities (Lanning, 1987; Stanovich, 1999).

The results presented in this study have certain clinical applications aimed at treating symptoms of schizophrenia. It has been shown that greater deviations in probability estimates occur in ambiguous scenarios. Precisely in highly ambiguous contexts decisions needs to rely more on top-down processes, to be more controlled and more guided by executive processes. With less ambiguity, responses can be based on more automatic estimations. Many of the results presented above can be interpreted as lack of cognitive control. So, it provides guides on where to start cognitive rehabilitation. In this sense, it has been argued that getting additional evidence against an illusion does not abolish it (Hohwy, 2013). Therefore, it is necessary to develop cognitive-behavioral interventions to train patients to collect and consider the whole evidence available to make better decisions. This would probably have an impact on the clinical symptomatology.

To the best of our knowledge, the present study is the first one to examine in a within-subject design the variation of participants’ responses to the Beads task across different levels of uncertainty and type of content and including facial expressions of emotions. Our version of the task have been shown to be sensitive to the way in which the ambiguous scenarios are interpreted as well as to emotion-related interpretative biases. Regarding the limitations of this study, it is worth noting that the sample of patients with schizophrenia were hospitalized (close to the discharge), which implies that symptoms were exacerbated. Additionally, cognitive deficits and other alterations that may impact task performance have not been controlled (Freeman et al., 2014; Tripoli et al., 2015).

In conclusion, results suggest that even though healthy controls show a certain deviation from the normative model, patients with schizophrenia deviate to a greater extent, especially under uncertainty scenarios. Those deviations from the normative model would reflect a failure to update beliefs in the face of new evidence as well as a difficulty integrating new evidence into existing knowledge. Also, the severity of delusions and the grade of disruption they cause to patients are correlated with impairments in data gathering with neutral and emotional content, respectively. These results could be used to inform cognitive therapeutic approaches of schizophrenia.

## Data availability statement

The raw data supporting the conclusions of this article will be made available by the authors, without undue reservation.

## Ethics statement

The studies involving human participants were reviewed and approved by Comité de Ética de la Investigación Clínica con Medicamentos CEIm, Hospital 12 de Octubre. The patients/participants provided their written informed consent to participate in this study.

## Author contributions

VR-F, RS, and JA designed the study. VR-F, PM-B, PR-G, EM, and NM-G managed the literature search and review. ES-M and RR-J selected the sample and evaluated patients. VR-F, RS, JA, and CR undertook the statistical analysis. VR-F, PM-B, EM, NM-G, and RR-J wrote the first draft of the manuscript. All authors contributed to the article and approved the submitted version.

## Funding

This work was supported in part by the Spanish Ministerio de Ciencia, Innovación y Universidades (grant number PSI2018-098876-B-100) to EM and in part by the Instituto de Salud Carlos III (grants PI16/00359, PI18/1275, PI19/00766; Fondo de Investigaciones Sanitarias/FEDER), by the European Development Regional Fund ‘‘A way to achieve Europe’’ (ERDF), by Madrid Regional Government [R&D activities in Biomedicine S2017/BMD-3740 (AGES-CM 2-CM)] and Structural Funds of the European Union.

## Conflict of interest

RR-J has been a consultant for, spoken in activities of, or received grants from: Instituto de Salud Carlos III, Fondo de Investigación Sanitaria (FIS), Centro de Investigación Biomédica en Red de Salud Mental (CIBERSAM), Madrid Regional Government (S2010/ BMD-2422 AGES; S2017/BMD-3740), JanssenCilag, Lundbeck, Otsuka, Pfizer, Ferrer, Juste, Takeda, Exeltis, Angelini, Casen-Recordati.

The remaining authors declare that the research was conducted in the absence of any commercial or financial relationships that could be construed as a potential conflict of interest.

## Publisher’s note

All claims expressed in this article are solely those of the authors and do not necessarily represent those of their affiliated organizations, or those of the publisher, the editors and the reviewers. Any product that may be evaluated in this article, or claim that may be made by its manufacturer, is not guaranteed or endorsed by the publisher.
