# Supplementary material for: Bayesian reasoning with emotional material in patients with schizophrenia
Source: Front Psychol. 2022 Nov 3;13:827037. doi: 10.3389/fpsyg.2022.827037 (PMC9670539; doi:10.3389/fpsyg.2022.827037)
Supplement: Supplementary file 1 [file Data_Sheet_1.docx]

Supplementary Material

**Appendix A**

*Calculation of difference from the ‘ideal observer’*

First, the exact probabilities following the Bayes theorem were calculated referring to jar B: being *p* the proportion of red beads in jar B, the probability of drawing a red bead (*r*) given that the jar of origin is B is P(*r*|B) = p. In the same way, for jar A, the probability of drawing a green bead (*g*) is P(*g*|A) = p, as long as the ratio of beads between jars is kept symmetrical. Prior probabilities of beads coming from either jar A or B before any bead is drawn are the same, P(B) = P(A) = 0.5. We consider the following notation: *n_d_* is the number of draws that have taken place and *n_r_* is the number of them that are red, and so *n_d_ - n_r_* is the number of them that are green. Then, P(B) = P(B| *n_d_* = 0*, n_r_* = 0) = 0.5, and the same for P(A). Following the Bayes theorem, the posterior probability of jar B being the underlying cause if *r* red beads and *g* green beads have been drawn is:

P(B| *n_d_, n_r_*) =$\frac{p^{n_{r}}\cdot(1-p)^{n_{d}-n_{r}}}{p^{n_{r}}\cdot(1-p)^{n_{d}-n_{r}}+(1-p)^{n_{r}}\cdot p^{n_{d}-n_{r}}}$ [1]

By means of this formula, responses from an "ideal observer" were calculated. Thus, the final score was the absolute value of the difference between individuals' responses (Y) and that of the ‘ideal observer’. Higher values implied greater deviation from the normative model. Then,

Difference from the ‘ideal observer’ = |Y-10·P(B| *n_d_, n_r_*)|

**Appendix B**

As mentioned in the statistical analysis section, the estimation-equation models allow working with different probability distributions. After testing several families of continuous probability density functions as well as link functions, the most appropriate was the gaussian family with identity link. Once these parameters were established, different correlation structures were tested using the quasi-likelihood under the independence model criterion (QIC) (Pan, 2001). Using the *model.sel* function from the MuMIn package (Barton, 2009), the best option (smaller QIC) was the first-order autoregressive (AR(1)) correlation structure. Once the correlation structure was stablished, non-significant (*p* >.1) interactions and factors were sequentially removed from the model. Then, the complete model was compared to the reduced one using the *anova* function from the geepack package. In all cases, the reduced model was significantly better than the complete model. Finally, post-hoc comparisons were tested using the type III anova from the *lsmeans* function from the homonym package (Lenth, 2016). The complete model, which included at the beginning the same variables and interactions for every ratio was as follows:

CompleteModel <- geeglm(DV ~ Group + Bead + Series + Content + Group*Content + Group*Series + Group*Bead+ Group*Series*Bead, Group*Series*Content, id=Subject, family = gaussian(link = identity), corstr="ar1", data= data.frame, scale.fix=F)

Barton, K. (2009). MuMIn: multi-model inference. *R Packag.*

Lenth, R. (2016). Least-squares means: the R package lsmeans. *J. Stat. Softw.* 69, 1–33.

Pan, W. (2001). Akaike’s Informat ion Criterion in Generalized Estimating Equations.

Supplementary Table 1

Summary of the proportion of beads and faces provided on each series in the presence of jar ratios of 60:40 and 80:20. R=red; G=green; H=happy; A=angry.

| **Series** | **Jar ratio of 60:40** | |  | **Series** | **Jar ratio of 80:20** | |
| --- | --- | --- | --- | --- | --- | --- |
|  | Beads | Faces |  |  | Beads | Faces |
| 1 | 60R:40G | 60H:40A |  | 6 | 80R:20G | 80H:20A |
| 2 | 100R:0G | 100H:0A |  | 2 | 100R:0G | 100H:0A |
| 3 | 50R:50G | 50H:50A |  | 3 | 50R:50G | 50H:50A |
| 4 | 40R:60G | 40H:60A |  | 7 | 20R:80G | 20H:80A |
| 5 | 0R:100G | 0H:100A |  | 5 | 0R:100G | 0H:100A |
